# Determinants of stunting in children aged between 6–23 months in Musanze region, Rwanda

**DOI:** 10.3389/fnut.2022.1044350

**Published:** 2022-11-24

**Authors:** Nadine Umwali, Catherine Nkirote Kunyanga, Dasel Wambua Mulwa Kaindi

**Affiliations:** Department of Food Science, Nutrition and Technology, University of Nairobi, Nairobi, Kenya

**Keywords:** infant and young child feeding practices, nutritional status, stunting, risk factors, minimum acceptable diet

## Abstract

Under-nutrition causes approximately half of all deaths in young children every year globally which is exacerbated by the multiple malnutrition burden. Infant and young child feeding practices pose immediate effects on the nutrition status of under 2 years aged children and greatly influence the survival of a child. This study aimed at determining the implication of the infant and young child feeding practices in evaluating stunting in young children among other stunting risk factors. Analytical cross-section study was carried out in Musanze, a district of Rwanda and involved 241 mothers having children aged between 6 and 23 months. Data was collected using a validated semi-structured questionnaire with observations and check list guides. Chi-square test and logistic regressions were used to determine the associations and risk factors of various variables. The results show that minimum meal frequency (MMF) was attained at 83% rate, minimum dietary diversity (MDD) at 57%, minimum acceptable diet (MAD) at 53% with consumption of iron rich foods at 29%. Stunting prevalence was 28%. The MAD had a significant (*p* = 0.021) association with height-for-age Z-score of a child and was found to be the stunting's predictor. The child's sex, consumption of animal sourced foods, child underweight status and income type were revealed as other stunting risk factors. A holistic approach that promotes infant and young child feeding practices and complementary feeding in particular can contribute to the alleviation of the stunting burden in Rwanda. Further, other associated factors that influence child nutrition status should be taken into consideration by the policy decision makers and development partners when developing food and nutrition sensitive programs and interventions.

## Introduction

Recently, the world's hunger situation has been threatened by the ravages of COVID-19 pandemic, conflict, and climate change and last year the projections by the Food and Agriculture Organization of the United Nations (FAO) reported 657 million people will be undernourished in 2030 which is nearly 30 million more than if the pandemic had not happened (1). Food insecurity is complex challenge in most global South countries and it has been reported that about 81% percent of all households in Rwanda are food secure with 39% out of this being considered marginally food secure and 19% percent are food insecure whereby out of these, 1.7% are severely food insecure (2). The Global Hunger Index (GHI) Scores by 2021 in the GHI rank placed Rwanda at 26.4 which is a serious state with 35.2% of the population being undernourished, and a high prevalence of stunting for children under 5 years at 33.1% (3). Malnutrition is recognized as a major universal concern that has various forms and can affect anyone in the world at certain point in life, despite of one's age, sex, wealth or geographical area (4). Although all people can suffer from malnutrition, young children are among the most affected (5). Undernutrition is estimated to be associated with 2.7 million child deaths annually or 45% of all child deaths (4).

The first 2 years of a child's life are particularly important, as optimal nutrition during this period lowers morbidity and mortality, reduces the risk of chronic disease, and fosters better overall development. Infant and young child feeding (IYCF) practices possess immediate effects on the nutritional status of under 2 years aged children and greatly influence the survival of a child (6). WHO (7) recognizes promoting proper IYCF practices as being one of the most successful interventions in ameliorating the health of a child and reports that potential growth and development are attained when children are fed properly especially those in the critical window of 0 and 24 months of age (8). IYCF indicators such as exclusive breastfeeding, minimum meal frequency (MMF), minimum dietary diversity (MAD) and minimum acceptable diet (MAD) have been largely associated with nutritional status outcome of children (9, 10). After analyzing statistics on IYCF practices globally, UNICEF (8) emphasized on the urgent development of programs in this area and specially showed considerable need for improving how children in complementary feeding period (6–23 months) are fed. The statistics were showing that feeding children aged 6–23 with WHO recommended minimum meal frequency (MMF), minimum dietary diversity (MDD) and minimum acceptable diet (MAD) were done at 51, 25, and 16% rate, respectively.

Few children receive nutritionally adequate and safe complementary foods; in many countries less than a fourth of infants 6–23 months of age meet the criteria of dietary diversity and feeding frequency that are appropriate for their age (7). Over 820,000 children's lives could be saved every year among children under 5 years, if all children 0–23 months were optimally breastfed (11).

The current Demographic Health Survey (DHS) aggregated statistics in Rwanda for under 5 years children is 33.1% for stunting levels and wasting at 1.1% (12) with the stunting levels for 6–8 months, 9–11 months, 12–17 months, and 18–23 months reported at 18.2, 21.3, 41.6 and 49.4%, respectively. In Rwanda, only 22 % (12) children of age between 6 to 23 months adhere to the infant young child feeding practices in terms of minimum acceptable diet, despite the high stunting prevalence that is very high (stunting ≥30%) according WHO threshold (13). Few children receive nutritionally adequate and safe complementary foods; in many countries less than a fourth of infants 6–23 months of age meet the criteria of dietary diversity and feeding frequency that are appropriate for their age (7). Breastfeeding improves IQ, school attendance, and is associated with higher income in adult life but only 37% of children younger than 6 months of age are exclusively breastfed in low-income and middle-income countries (14). Dewey and Begum (15) have reported that being stunted is a risk factor for reduced survival, childhood and adult health, and reduces the capacity of learning and production. Therefore, improving child development and reducing health costs through breastfeeding results in economic gains for individual families as well as at the national level. It is against this background that this study sought to find out whether IYCF practices play role in determining the stunting level in Rwanda.

## Materials and methods

### Study design and setting

The study was conducted in Northern Province of Rwanda, in the District of Musanze. A cross-sectional study design was used for data collection in the study sites. The study took place at nine health centers purposively selected from 16 health centers found in Musanze District. The district was purposely chosen since it is food secure district and yet has a high stunting prevalence of 38%. The study targeted the mothers having children aged of 6–23 months from which 241 mothers fulfilling all the study inclusion criteria participated in the study.

### Sample size determination

The sample size was calculated using the formula of Fischer et al. (16) where the prevalence of infant and young child feeding practices in Rwanda (18%) was used as *p*-value. The sample size was calculated using formula of Fischer et al. (16) as follows:


$$n=z^{2}pq/d^{2}$$

Where

n= the desired sample size when population is >10,000

z= the standard normal deviation which is 1.96 at 95 % confidence interval

p= prevalence of IYCF practices 18 %, (Demographic and Health Survey (2014/15)

q=1-p=1-0.18=0.82

d= the degree of accuracy desired set at 5 % (0.05)

Therefore;

n=1.96^2^*0.18^*^0.82=227/0.05^2^

5.5 % attrition=227/0.945=241 (attrition = 14)

Therefore, the total sample size =241.

### Sampling procedure

**Figure 1:**
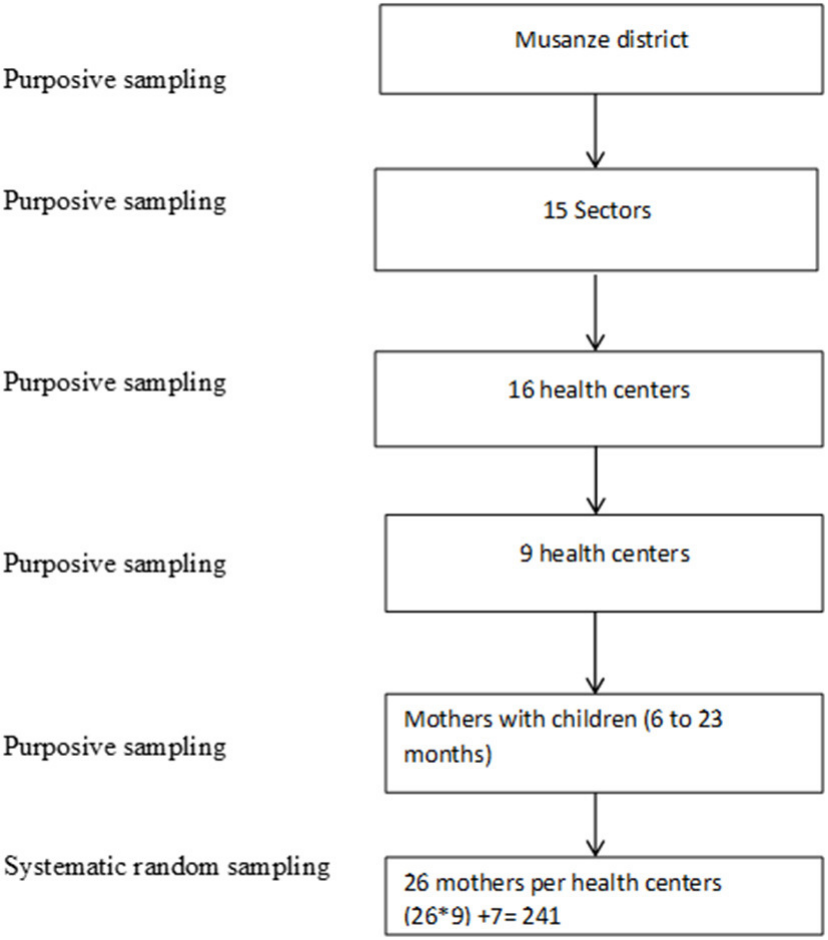
Sampling schema of the study.

The sampling schema is shown in Figure 1. Musanze District was sampled purposely for the study as it has a high stunting prevalence of 37.8 % despite being 80% food secure according to CARI index (17). The district is divided in 15 sectors with 16 health centers. The data were collected in 9 health centers purposively selected based on the ones having higher cases of acute malnutrition. The health centers were Nyakinama, Nyange, Kimonyi, Kinigi, Rwaza, Karwasa, Muhoza, Gataraga and Musanze. The study participants were the mothers having children aged between 6 and 23 months who had visited the health center at the time of data collection. Systematic random sampling was used to select 27 mothers from each of the nine health centers.

#### Inclusion criteria

All mothers having children of 6–23 months old who had attended the selected nine Health Centers and who gave consent.

#### Exclusion criteria

The children who had severe or critical illness or referral cases were excluded from the study.

### Data collection tool and procedure

#### Data collection

Quantitative research method was used in the study. By using a semi-structured questionnaire, the information on socio demographic and socioeconomic characteristics were collected; age, sex, education level of the mothers, household size, marital status, household occupation, income, land ownership, and wealth category. The information was obtained by face-to-face interviews with the mothers of the children under the study and data were analyzed using the descriptive statistics. Observational study was also employed during the interviews. Further, a pre-tested semi-structured questionnaire was used to collect data on maternal nutrition knowledge, IYCF practices, nutrition information network and anthropometric measurements. The questionnaire was administered to the mothers and each response given was filled well in its respective section.

In Rwanda, all households are classified into categories called “Ubudehe,” which is a social stratification programme depending on income among households, thus reflecting their economic status. The study used the classification done into 2015 where the population was put into 4 Ubudehe categories based on the resources and assets owned by households as well as the ability of sustaining their livelihoods. The categories are the first (poorest), second, third and fourth (richest) Ubudehe categories (18, 19). In this study, these categories are referred as wealth categories. During the data collection, every mother under the study was asked the category in which her household had been classified and the answer was recorded.

#### Twenty-four hour dietary recall

In order to capture all the foods, beverages and the frequency at which children were fed in 24 h preceding the interview, a 24- h dietary recall questionnaire was used which collected data on the time of the food consumption, the name and ingredients of the dish followed by the corresponding quantities.

#### Anthropometric measurement

The anthropometric measurements were taken referring to WHO (20) recommended guidelines for measuring weight and height (length) of under 2 years children. For height, the UNICEF height board was used to measure the length of the child and read to the nearest 0.1 cm. In the first place, the mother helped to take off the excess clothes and shoes of the child prior to measuring. Then, the height board was horizontally placed on a flat and leveled surface and the height (length) was obtained by the child lying on it straight with feet together, knees straight, heels and buttocks in contact with the board, the shoulders relaxed, arms straight at the sides and the shoulder blades touching the length board. The measurements were taken twice from which an average length was calculated. For the weight, an electronic SECA scale was used to measure the weight of the child. The scale was placed on flat and stable surface and checked for accuracy and verified using an object of known weight before every weighing session. Children only remained with lightweight clothes (without jackets, socks and shoes). The measurements were taken twice from which an average weight was calculated and reported to the nearest 0.1 kg.

### Data quality control

#### Pretesting of study tools

The questionnaire was pre-tested on 20 mothers in a selected pilot health facility before commencing actual data collection to ensure familiarization of the field assistants with the questionnaire, equipment, obtaining consent, checking on its validity and find out if it would respond to objectives of the study.

#### Recruitment and training of field assistants and enumerators

The recruitment of field assistants and enumerators was advertised verbally within the study district. The criteria for recruitment consisted of good conduct and reliability, attainment of college education, having basic nutritional knowledge, experience in data collection, and preferably being a resident of the study area. The shortlisted candidates were interviewed. The training took 4 days and the covered subjects included the study objectives, the use of survey equipment, interviewing techniques, anthropometric measurements, and filling the questionnaire. The whole team went through the questionnaire to understand its contents for uniformity in interpretation of the questions. They were trained as well on good behavior and courtesy while interacting with and interviewing the participants.

#### Weight scale assurance, and length quality assurance mechanism

The scale was placed on flat and stable surface and checked for accuracy and verified using an object of known weight before every weighing session. Children only remained with lightweight clothes (without jackets, socks and shoes). For the length quality assurance, the height board was horizontally placed on a flat and leveled surface and the height (length) was obtained by the child lying on it straight with feet together, knees straight, heels and buttocks in contact with the board, the shoulders relaxed, arms straight at the sides and the shoulder blades touching the length board. For both anthropometries, length and weight, measurements were taken twice from which an average length was calculated.

#### Ethical consideration

Ethical clearance certificate was sought from Rwanda National Ethics Committee (reference no. 681/RNEC/2019) as well as a written approval from Musanze district administration. The informed consent was also sought from health centers administration and the study participants were interviewed after signing the informed consent form.

### Assessment of infant and young child feeding practices of children aged 6–23 months

The assessed infant and young child feeding practices' indicators include minimum meal frequency (MMF), Minimum dietary diversity (MDD) and Minimum acceptable diet (MAD). The tools used were developed following the guidelines recommended by FAO (21). The tools included a semi-structured questionnaire composed of questions reflecting on the pre-mentioned indicators; a seven food-groups dietary diversity checklist as well as a 24-h dietary recall questionnaire where the mothers indicated all foods and drinks the child consumed 24 h before the start of the survey. The MMF was calculated based on the number of meals (solid, semi-solid or soft foods) fed to the child during the 24 h preceding the interview. The criteria for meeting the MMF recommended by WHO vary depending on the age and the breastfeeding status of the child (22). Among the breastfed children, receiving at least two meals (when aged of 6–8 months) or at least three meals (when aged of 9–23 months) were the conditions to achieve the MMF. Regardless of the age, the non-breastfed children had to be fed at least four times the previous day to be classified as having met the MMF. The recommended MDD was calculated referring to WHO/UNICEF (22) guidelines. A seven food groups checklist was used to determine the individual dietary diversity score (IDDS) reflecting the number of food groups a child was fed from 1 day before the interview. The food groups on the checklist were grains, roots and tubers; legumes and nuts; dairy products; flesh foods; eggs; vitamin A fruits and vegetables and other vegetables and fruits. The conditions for meeting MDD differ for breastfed children and non-breastfed children (22). Consequently, being fed from four food groups or more was a criterion to achieve the MMD among breastfed children whereas the consumption of at least four food groups, without including the milk feeds, was a condition for non-breastfed children. Children met the MAD when they had achieved at the same time the MMF and MDD 24 h before the survey. The consumption of at least 2 milk feeds was an added condition to non-breastfed children for them to achieve the MAD (22).

### Data processing and analysis

#### Nutritional status of children

By using height/ length boards and electronic SECA scales as tools and referring to WHO guidelines, two anthropometric data were taken, namely, height (length) and weight, for children of 6 to 23 months of age (23). The nutritional status was assessed by determining the three nutrition indicators namely; stunting, wasting and underweight. A Height-for-age Z-score (HAZ), weigh-for-height Z-score (WHZ) and weight-for-age Z-score (WAZ) that fell under a minus two standard deviation (SD) indicated the state of stunting, wasting and underweight, respectively.

#### Data analysis

Statistical Package for Social Science (SPSS) software version 20.0 was used for data entry, cleaning, and analysis with different statistical tests whereas anthropometric data were analyzed using Emergency Nutrition Assessment software (ENA for Smart 2014, https://smartmethodology.org/wp-content/uploads/2014/11/ENA-Manual.pdf) to determine different nutritional status of children. Descriptive statistics were used for socio-demographic and economic, maternal knowledge, feeding practices and nutritional status data analysis. Chi-square and the independent *t*-test were used to determine the association between different IYCF practices and nutritional status of children. Binary logistics regression model was used to determine factors influencing stunting.

## Results

### Socio-demographic profile of participant households

The socio-demographic and socio-economic profiles of the study participants are showed in Table 1. The findings show that the mean household size was 4.7 (SD = 1.8) with a minimum of two (1) members. The mean age of the index children was 11.8 months (SD = 4.5). The mean age of the mothers was 29 years with the oldest interviewed mother being 50 years old. Most of the mothers (56.4%) have attained primary school education with 27% attending secondary school and only 4.1 % attended University. Nearly 12.4% of mothers had no formal education. The study revealed that farming and casual labor were the two predominant sources of income of the households, 38 and 37%, respectively. The households that were found to possess land for food production were 68% whereas 32% of the households did not produce food. The results on the household's classification into wealth categories indicated that majority of the respondents were in the second wealth category (54%). The study found that only a few respondents (17%) were in the first wealth category whereas 30% were in the third wealth category. It is noteworthy that no household was found to be in the fourth wealth category, the richest category. The mean nutrition knowledge score was found to be 71.03 (15.06 SD) and the majority (72%) of the mothers had a high (score ≥70% score) knowledge score whereas 28% had a low (score < 70%) knowledge score.

**Table 1:** Sociodemographic and socio-economic profile of households (*n* = 241).

**Variable**	**Pooled *n* = 241**		**1^st^ wealth category *n* = 40**		**2^nd^ wealth category *n* = 130**		**3^rd^ wealth category *n* = 71**		**Chi2**	***p*-Value**
	**Freq**	**%**	**Freq**	**%**	**Freq**	**%**	**Freq**	**%**		
**Marital status of mothers**									18.33	0.005^***^
Married	206	85.5	29	72.5	113	87	64	90		
Single	22	9.1	5	12.5	12	9	5	7		
Separated	3	1.2	3	7.5	0		0			
Widowed	10	4.2	3	7.5	5	4	2	3		
**Education of mothers**									49.31	0.000^***^
No formal education	30	12.4	12	30	12	9.2	6	8.5		
Some primary	69	28.6	21	52.5	32	24.6	16	22.5		
Completed primary	67	27.8	2	5	44	33.8	21	29.6		
Some secondary	37	15.4	22	12.5	10	16.9	37	14.1		
Completed secondary	28	11.6	0		18	13.8	10	14.1		
University/college	10	4.1	0		2	1.5	8	11.3		
**Number of under five children per HH**									4.745	0.314
1	161	66.8	23	57.5	91	70.0	47	66.2		
2	76	31.5	15	37.5	38	29.2	23	32.4		
3	4	1.7	2	5.0	1	0.8	1	1.4		
**Sex of index child**									1.227	0.541
Male	115	47.7	16	40	65	50	34	47.9		
Female	126	52.3	24	60	65	50	37	52.1		
**Religion of HH**									19.85	0.001^***^
Catholic	127	52.7	10	25	82	63.1	35	49.3		
Protestant	112	46.5	29	72.5	48	36.9	35	49.3		
Muslim	2	0.8	1	2.5	0	0.0	1	1.4		

*** Represents significance at 1% level.

### Socio-economics characteristics of households

Majority of the mothers (49%) were farmers, 21% casual labors, 9% business women while 17% were unemployed (Table 1). On the other hand, the predominant occupation of the heads of households was casual labor (39%), followed by farming (37%), salaried job (13%) and business (9%). Out 32 the salaried households head, 72% were from the third wealth category. Furthermore, the study revealed that farming and casual labor were the two predominant sources of income of the households, 38 and 37%, respectively. The households that were found to possess land for food production were 68% whereas 32% of the households did not produce food (Table 2). The total household income for the month preceding the survey was below 10,000 Rwandan francs for the majority of the households (46%). Out of households which earned more than fifty thousand Rwandan francs, 61% were from third wealth category and only 2% from first wealth category.

**Table 2:** Socio-economic characteristics of household members across their wealth categories.

**Variable**	**Pooled *n* = 241**		**1^st^ category *n* = 40**		**2^nd^ category *n* = 130**		**3^rd^ category *n* = 71**		**Chi2**	***p*-Value**
	**Freq**	**%**	**Freq**	**%**	**Freq**	**%**	**Freq**	**%**		
**Occupation of mothers**									37.601	0.000^***^
Salaried job	9	3.7	0		1	0.8	8	11.3		
Farmer	117	48.5	15	37.5	70	53.8	32	45.1		
Business	21	8.7	2	5	9	6.9	10	14.1		
Casual labor	50	20.7	18	45	24	18.5	8	11.3		
Crop/animal sales	2	0.8	0	0	1	0.8	1	1.4		
Housewife	1	0.4	0	0	1	0.8	0			
Unemployed	41	17	5	12.5	24	18.5	12	16.9		
**Occupation of HHH**									43.537	0.000^***^
Salaried job	32	13.3	1	2.5	8	6.2	23	32.4		
Farmer	89	36.9	13	32.5	53	40.8	23	32.4		
Business	21	8.7	1	2.5	13	10.0	7	9.9		
Casual labor	94	39	24	60	54	41.5	16	22.5		
Crop/animal sales	1	0.4	0	0.0	0	0.0	1	1.4		
Unemployed	4	1.7	1	2.5	2	1.5	1	1.4		
**Major source of income of the HH**									47.030	0.000^***^
Salaried job	30	12.4	1.0	2.5	6	4.6	23	32.4		
Farmer	92	38.2	13.0	32.5	55	42.3	24	33.8		
Business	21	8.7	3.0	7.5	11	8.5	7	9.9		
Casual labor	89	36.9	22.0	55.0	53	40.8	14	19.7		
Casual trade	7	2.9	0.0	0.0	4	3.1	3	4.2		
Remittance/gift	2	0.8	1.0	2.5	1	0.8	0	0.0		
**Total HH income in the last month (Rwf)**									29.415	0.000^***^
<10,000	110	45.6	25	62.5	63	48.5	22	31.0		
10,000–20,000	45	18.7	7	17.5	28	21.5	10	14.1		
20,000–30,000	18	7.5	4	10.0	9	6.9	5	7.0		
30,000–50,000	29	12.0	3	7.5	16	12.3	10	14.1		
50,000 and above	39	16.2	1	2.5	14	10.8	24	33.8		
**HH access to the land for food production**									0.584	0.747
Yes	163	67.6	25	62.5	89	68.5	49	69		
No	78	32.4	15	37.5	41	31.5	22	31		

*** Represents significance at 1% level.

### Child feeding practices among mothers

The results show that most of the children (79%) were fed at least 3 times the day before the survey whereas 2.5% did not consume any solid or semi solid food 1 day before the survey (Table 3). The mean IDDS score for all children was found to be 3.5 (1.25 SD) and the majority had the medium dietary diversity score (4–6 score), followed by the low dietary score (43%) (≤3 score). The proportions of 241 children under the study who achieved the MMF, MDD and MAD were 83%, 57 and 53%, respectively. The consumption of the animal sourced foods was at 28% rate (Table 3).

**Table 3:** Distribution of children (6–23 months) by the feeding practices (*n* = 241).

**Feeding practices**	**Frequency**	**Percent**
**Individual dietary diversity score (IDDS)**		
High dietary diversity (≥6 score)	9	3.7
Medium dietary diversity (4–5 score)	128	53.1
Low dietary diversity (≤3 score)	104	43.2
**Meal frequency**		
0 meal	6	2.5
1 meal	11	4.6
2 meals	34	14.1
3+ meals	190	78.8
**Consumption of iron rich foods**		
Yes	70	29
No	171	71
**Consumption of animal sources foods**		
Yes	68	28.2
No	173	71.8
**Meeting minimum meal frequency**		
Yes	199	82.6
No	42	17.4
**Meeting minimum dietary diversity**		
Yes	137	56.8
No	104	43.2
**Meeting minimum acceptable diet**		
Yes	128	53.1
No	113	46.9

### Nutritional status of children

Almost 28% of 241 children measured were stunted where 9% and 19% were severely and moderately stunted, respectively (Table 4). The male children had higher stunting rate than their female counterparts, 40 and 17%, respectively. Global acute malnutrition (wasting) prevalence was 2% where 1% of male children were wasted against 2% of female children. Out of 241 children under this study, 95% had a good nutrition status in terms of underweight, whereas the prevalence of moderate and severe underweight was found to be 4 and 1%, respectively.

**Table 4:** Distribution of children by their nutritional status (*n* = 241).

	**Males (%)**	**Females (%)**	**All (%)**
Stunting^*^			
Overall	40	16.7	27.8
Moderate	25.2	13.5	19.1
Severe	14.8	3.2	8.7
**Wasting**			
Global	0.9	2.4	1.7
Moderate	0.9	1.6	1.2
Severe	0	0.8	0.4
**Underweight**			
Overall	8.7	2.4	5.4
Moderate	6.1	2.4	4.1
Severe	2.6	0	1.2

* Stunting, wasting, underweight = < -2 Z score; Moderate = -3 < Z score < -2; Severe = < -3 Z score and/or oedema for wasting.

Using Chi-square test, the study revealed that the majority of children who met the IYCF practices had a good nutritional status (Table 5). The results show that 74, 73, 76 and 63% of children who met MMF, MDD, MAD and consumption of iron-rich foods, respectively, were not stunted. Moreover, 2, 3, 3 and 1% of children with recommended MMF, MDD, MAD and iron-rich food consumption, respectively, were wasted. Lastly, 6, 3, 8 and 10% who achieved MMF, MDD, MAD and consumption of iron-rich foods respectively, were underweight. However, when the significance tests were conducted between the above IYCF practices and different forms of malnutrition, the associations were found to be no significant at 95% Confidence Interval (Table 6). Nevertheless, the study revealed that the MAD has a statistically significant (*p* = 0.021) association with height-for-age Z-score (HAZ) of a child. The results of an independent sample t-test show a significant difference between the HAZ mean (−1 SD) of children who met the MAD and the HAZ mean (−1.5 SD) of those who did not (Table 6).

**Table 5:** Association of IYCF practices with nutritional status of children.

**IYCF practices**	**Pooled *n* = 241**	**Stunting**			**Wasting**			**Underweight**		
		**Yes**	**No**	**χ^2^**	**Yes**	**No**	**χ^2^**	**Yes**	**No**	**χ^2^**
MMF (yes)	199	26%	74%	0.20	2%	98%	0.35	6%	94%	0.34
MDD (yes)	137	28%	73%	0.98	3%	97%	0.07	3%	97%	0.13
MAD (yes)	128	24%	76%	0.18	3%	97%	0.05	8%	92%	0.08
Consumption of iron-rich foods (yes)	70	37%	63%	0.13	1%	99%	0.64	10%	90%	0.16

χ^2^: Chi-square (p-value).

**Table 6:** Independent *t*-test between HAZ score and MAD.

	**Did the child meet MAD?**				
**Variable**	**No**	**Yes**	**Std. Error difference**	**t-value**	***p*-Value**
HAZ score	−1.49331	−1.05326	0.189599	−2.321	0.021^*^

* Represents *p* < 0.05.

### Determinants of stunting

The determinants of stunting were identified by using the multiple logistic regression analysis. The results showed that the factors that predict stunting are MAD, income type, sex of the child, consumption of animal sourced foods and underweight status (Table 7). The findings revealed that there was a significant (*p* = 0.009) negative relationship between the child meeting the MAD and stunting. The household income type (farming) (*p* = 0.021), child underweight (*p* = 0.000) were found to have a significant negative relationship with stunting whereas the sex (male) of a child (*p* = 0.003) and consumption of animal sourced foods (*p* = 0.047) had a significant positive relationship with stunting.

**Table 7:** Determinants of stunting among children aged of 6–23 months.

**Variable**	**Coefficient (B)**	**Standard error**	**Sig**.
Income type (Farming HH = 1)	−0.946	0.411	0.021^*^
MAD (yes = 1)	−1.385	0.527	0.009^*^
Higher Education of the mother (yes = 1)	−0.659	0.426	0.122
Iron-rich foods consumption (yes = 1)	0.787	0.472	0.095
Age of the mother (above 35 years = 1)	−0.017	0.400	0.967
Consumption of animal sourced food (yes = 1)	−1.109	0.558	0.047^*^
HH Wealth category (2^nd^or higher = 1)	−0.165	0.462	0.721
Maternal nutrition knowledge (high = 1)	0.194	0.388	0.617
Meal frequency of a child	−0.005	0.195	0.982
IDDS of a child	0.468	0.258	0.070
Access to land (yes = 1)	0.752	0.423	0.075
Sex of a child (male = 1)	1.026	0.346	0.003^*^
Underweight status (yes = 1)	3.184	0.870	0.000^*^
Constant	−2.590	0.862	0.003

Dependent variable: Stunting.

* Prediction is significant.

## Discussion

The proportion of children who met the IYCF indicators are higher compared to the 2014-2015 RDHS report showing that in Northern Province, the rate of meeting the MMF, MDD and MAD was 54, 34 and 22%, respectively (24). This could be attributed to two main factors which are food security and maternal nutrition knowledge level in Musanze district. The 2018 Rwanda Comprehensive Food Security and Vulnerability Analysis (CFSVA) report indicates that, in Musanze district, the food security rate (by CARI index) has increased from 80% in 2015 to 88.5% in 2018. Additionally, many mothers (72%) demonstrated to have a high level of knowledge on feeding practices and 95% of respondents had been exposed to the nutrition education from different sources such as health centers, CHWs and community gatherings (groups). This is supported by a study conducted in Indonesia where the diet of children whose mothers had been exposed to nutrition information improved in terms of meeting MAD (25). Similar findings were observed in one of the studies done in Ethiopia where Berra and young (26) found that maternal knowledge on complementary feeding is one of the key determinants of suboptimal complementary feeding practices and is positively associated with meeting MMF and MAD among children aged between 6 and 23 months.

The stunting rate in this study is lower as compared to 38% rate reported in 2014-2015 RDHS report. This decrease could be explained by the time factor where the prevalence might have decreased as the years passed. The RDHS (2014-2015) report indicates the trend of malnutrition decreasing over the years where the stunting rate dropped from 51% in 2005 to 44% in 2010, then to 38% in 2015 (24). Those improvements may be attributable to the great effort done by the government of Rwanda through different strategies and programmes such as multisector participation and consensus around Rwanda's First National Nutrition Summit (2009), and Second National Nutrition Summit (2011), National Multi-Sector Strategy to Eliminate Malnutrition (2010), behavior change communication (including mass media), home food fortification by using micronutrient powders and First 1,000 Days Community Based Food and Nutrition Programs (24, 27) among many others.

In addition, the reduced stunting prevalence in the present study could be because larger proportion of children in the study met the MMF, MDD and MAD as compared to the children in 2014-2015 RDHS report as elaborated in the preceding paragraphs. This is supported by several researches that linked the nutritional status of children and child feeding practices. While assessing the association of IYCF indicators and stunting by reviewing the DHS data of different countries, Jones et al. (9) found that the odds of being stunted were significantly lower among the children (6–23 months) who had achieved the MAD (in Zimbabwe) and those who had met the MDD (in India). In Bangladesh, India, Zambia and Ethiopia, meeting the MAD was found to be associated with a higher Height-for-age Z-Score and there was a positive association between MDD and HAZ in those same countries except Ethiopia (9).

Concerning stunting determinants, the MAD was found to influence stunting negatively and significantly, implying that children who achieve the MAD are less likely to be stunted. Since meeting the MAD reflects the consumption of a significant number of meals and more diversified foods, children who meet this IYCF indicator are more likely to meet adequate nutrients required for child optimal development and growth (11), hence the prevention of stunting. This study is consistent with Jones et al. (9) who found that in Zimbabwe, the odds of being stunted were significantly lower among the children (6–23 months) who had achieved the MAD.

Though the root causes have not yet been clearly established, a considerable number of studies have asserted that male children are more likely to be undernourished than female children. The examples of these studies include Medhin et al. (28), Wamani et al. (29), Bork and Diallo (30), Mya et al. (31) and Sultana et al. (32). Also, this study linked stunting with the child's sex by finding that being a male child is associated with a higher chance of being stunted than a female child. Though, the reason behind this finding was not in the scope of this study, some researchers, such as Wells (33), claim that natural selection might be the cause of male children being more prone to infectious diseases and malnutrition in early stage of life as compared to girls. Moreover, Bork and Diallo (30) while conducting a study in rural Senegal, found that male children are introduced to early complementary feedings (before age of 6 months) which might be detrimental to their height status, probably resulting in having poorer nutrition status as compared to female children. According to Michaelsen et al. (34), introducing the complementary food before age of 6 months results in increased child morbidity and interferes with the bioavailability of breastmilk nutrients, hence gaining the potential weight and height by a child is likely reduced.

The consumption of animal sourced foods was another factor revealed by this study to negatively influence the stunting, implying that children who consume animal sourced foods are less likely to be stunted. This could be attributed to the fact that animal sourced foods such as meat, fish, eggs and dairy products are scientifically proven to contain high quality protein which according to Headey et al. (35), has been linked to the child growth by several nutritional researchers. Consequently, Dewey (36) recommends the daily inclusion of animal sourced foods in the complementary food for the child's insurance of meeting all nutrient needs. The present study's finding is in line with the research conducted in 46 countries (Asia, Africa, and Latino America) that concluded that the consumption of foods from animal origin is strongly associated with child growth, especially the milk products and fish (35). The similar results were found by Krasevec et al. (10) in study conducted in low- and middle-income countries.

Underweight was found to be significantly and positively associated to stunting. This implies that a child who is underweight has a bigger chance to become stunted. This finding was not surprising because stunting is defined as low height for age whereas underweight stands for low weight for age (4). Therefore, factors that can interfere with the child's optimal growth by affecting the weight, can easily affect the height as well. This is upheld by WHO (4) stating that an underweight person can suffer from stunting, wasting or both concurrently. The present finding is in consistent with Ngwira et al. (37) who found a significant association between underweight and stunting among under five children in Malawi.

Farming as major source of household income was found to be negatively associated with stunting, implying that in Musanze district, children belonging to households that farm as their main source of income are less likely to be stunted. This could be because farming increases the availability of and access to food items which in turn improves food and nutrition security of the households, thus the likelihood to reduce malnutrition. Moreover, the present study shows that 100% of households farming as major source of income own the land for crops production and according to 2018 Rwanda CFSVA report, land ownership among the agricultural households contributed to food security and more severe food insecurity was observed in households who did not own land as compared to those who owned land (2). A study done in India asserted a relationship between household food security and child undernutrition by finding that children from severely food insecure households have higher chances of suffering from severe stunting and underweight (38). The similar findings were as well found by Ali et al. (39).

## Data availability statement

The raw data supporting the conclusions of this article will be made available by the authors, without undue reservation.

## Ethics statement

The studies involving human participants were reviewed and approved by Ethical clearance certificate was sought from Rwanda National Ethics Committee (reference no. 681/RNEC/2019) as well as a written approval from Musanze district administration. The informed consent was also sought from health centers administration and the study participants were interviewed after signing the informed consent form. The patients/participants provided their written informed consent to participate in this study.

## Author contributions

NU and CK were involved in the conceptualization of the study. CK and DK were involved in general supervision of the study and project administration and assisted in the study proposal write up, ethical approval process follow up, survey tools development, data collection, and analysis methodology. All authors contributed in writing the original draft preparation, reviewing and editing, and read and agreed to the published version of the manuscript.

## Conflict of interest

The authors declare that the research was conducted in the absence of any commercial or financial relationships that could be construed as a potential conflict of interest.

## Publisher's note

All claims expressed in this article are solely those of the authors and do not necessarily represent those of their affiliated organizations, or those of the publisher, the editors and the reviewers. Any product that may be evaluated in this article, or claim that may be made by its manufacturer, is not guaranteed or endorsed by the publisher.
